# Racial Differences in Breastfeeding Initiation Among Participants in a Midwestern Public Health District

**DOI:** 10.1089/heq.2018.0016

**Published:** 2018-10-19

**Authors:** Maria Pineros-Leano, Karen M. Tabb, Shannon D. Simonovich, Yang Wang, Brandon Meline, Hsiang Huang

**Affiliations:** ^1^ School of Social Work, Boston College, Chestnut Hill, Massachusetts.; ^2^ IDEA Research Team, University of Illinois, Urbana, Illinois.; ^3^ School of Social Work, University of Illinois at Urbana-Champaign, Urbana, Illinois.; ^4^ School of Nursing, College of Science & Health, DePaul University, Chicago, Illinois.; ^5^ Maternal and Child Health Division, Champaign-Urbana Public Health District, Champaign, Illinois.; ^6^ Department of Psychiatry, Cambridge Health Alliance, Harvard Medical School, Cambridge, Massachusetts.

**Keywords:** breastfeeding initiation, low-income women, race/ethnicity, WIC

## Abstract

**Purpose:** Although variations in breastfeeding initiation are well documented, the contributing role of maternal race remains poorly understood, especially among the multiracial—two or more races—population. The purpose of this study is to examine differences in breastfeeding initiation among a racially and ethnically diverse population of low-income women.

**Methods:** Participants for this study (*n*=1010) were enrolled in the supplemental nutrition program for women, infant, and children and concurrently enrolled in a perinatal depression registry at a public health clinic in the Midwest. Race was obtained from medical records. Breastfeeding initiation was gathered through a clinical interview during the first postpartum visit. Logistic regression was conducted using STATA 14.2.

**Results:** Sixty-eight percent of study participants reported breastfeeding initiation. The bivariate analysis demonstrated that there were significant differences in rates of breastfeeding initiation by race/ethnicity. The logistic regression models showed that after adjusting for maternal education, age, income, nativity, parity, body mass index, and antenatal smoking, Black (odds ratio [OR] 0.47; confidence interval [95% CI] 0.34–0.66), multiracial (OR 0.21; 95% CI 0.07–0.65), and Latina women (OR 0.48; 95% CI 0.26–0.86) were significantly less likely to initiate breastfeeding compared with White women.

**Conclusion:** These findings highlight the need for further understanding of the underlying barriers to the initiation of breastfeeding among low-income Black, multiracial, and Latina women. Moreover, breastfeeding should remain a priority for intervention and policy development, particularly among racially and ethnically diverse low-income women.

## Introduction

Breastfeeding has multiple benefits for children, including reducing instances of infant mortality,^1^ diarrhea,^2^ allergies,^3^ asthma,^3^ childhood overweight and obesity,^4^ and leukemia.^5^ Breastfeeding benefits not only children, but also mothers.^6–8^ For instance, women who breastfeed are less likely to develop breast cancer,^7,9^ cardiovascular disease,^6^ and type 2 diabetes,^8,10^ and they are more likely to return to their prepregnancy weight.^11^ Considering all the advantages of breastfeeding for child and mother, the *Healthy People 2020* goal for breastfeeding initiation is 82%.^12^ Despite the current overall population-level initiation rate of 79.2%,^13^ the documented initiation rates for low-income women are only 57%,^14^ making it difficult to reach the goal among this population.^15,16^

Since rates of breastfeeding initiation vary among specific populations of women,^17^ many studies attempt to better understand the characteristics that prevent initiation. Some studies have found that women of low socioeconomic status (SES) and with lower education levels are less likely to breastfeed, compared with women of middle SES and with higher levels of education.^18^ Given these social characteristics, more attention has been devoted to investigate women who are part of the Women, Infant, and Children (WIC) program.^15^ WIC is a federally funded supplementary nutrition program for at-risk pregnant women and children under the age of 5. A study investigating breastfeeding rates among WIC participants found that Latina women had higher initiation rates and breastfed for longer duration compared with White and Black women.^15^ A recent study compared 3494 participants from Mississippi and found that White WIC participants were significantly less likely to initiate breastfeeding compared with White nonparticipants (50.8% vs. 70.8%).^19^ On the other hand, among Black women, breastfeeding initiation was not significantly different between participants and nonparticipants. It was also found that after adjusting for covariates, participation in WIC was negatively associated with initiation of breastfeeding only among White participants.^19^ Previous literature has also investigated the reasons behind lower breastfeeding rates among WIC populations.^20,21^ This literature has found that some of the main deterrents for breastfeeding include lack of social and professional support, and limited information about breastfeeding.^20,21^ In fact, a study found that attending prenatal peer support breastfeeding groups significantly increased breastfeeding initiation intentions, compared with those in the control group, demonstrating the importance of social and professional support.^22^

In addition to social characteristics, psychological determinants, especially antenatal depression, can impact breastfeeding initiation.^23^ Antenatal depression affects between 15% and 25% of women, but it particularly affects low-income women.^24,25^ A recent meta-analysis of 30 studies investigating the effects of maternal depression on perinatal outcomes found that women who had perinatal depression were less likely to initiate breastfeeding compared with those without maternal depression.^23^

Given the heightened vulnerability of WIC participants regarding breastfeeding and the need to better understand the role of antenatal depression, it is important to investigate the prevalence and correlates of breastfeeding across the United States (U.S.) among diverse racial/ethnic samples. The aim of this study is to identify breastfeeding initiation rates across racial/ethnic groups, and the correlates for breastfeeding initiation among WIC participants enrolled in a depression registry in a Midwestern city.

## Methods

### Design and setting

This is a cross-sectional study using data collected as part of a depression registry between April 2013 and October 2015. The sample came from a public health district with two clinics (one rural and one suburban). The clinics provide family case management, perinatal home visits, immunizations, and counseling to low-income women. In accordance with a state policy mandate, Maternal and Child Health case managers screen all expectant mothers for depressive symptoms using the Edinburgh Postnatal Depression Scale (EPDS) during the antenatal and postnatal periods.^26^

### Sample and procedures

All women receiving ongoing perinatal care and completing at least one EPDS mood questionnaire during their antenatal care were eligible for inclusion in the study. A letter inviting participation in the database registry was given to each potential participant at the time of intake. Trained research assistants consented respondents to participate in the study. In this study, all participants were also enrolled in WIC. Data were extracted from several sources: paper-based charts, electronic medical records, state vital records, and Cornerstone, the statewide WIC reporting system. All completed EPDS screens were filed in paper-based medical charts. To retrieve depression screen information, the study team pulled each chart for depression screen information.

Study procedures were approved by the Public Health Institute for Research and Excellence Review Committee and the University of Illinois at Urbana-Champaign Institutional Review Board.

### Measures

#### Breastfeeding initiation

Breastfeeding initiation was gathered through a clinical interview during the first postpartum visit, which usually took place within 1 week of the baby's birth. Clinicians asked participants whether they had put the baby to the breast at least one time after birth.

#### Race/ethnicity

Race and ethnicity were gathered from medical records. Race/ethnicity was classified into six different categories: Black, White, Asian, Latino/a, Multiracial, and Other. Multiracial was defined as two or more races self-reported at intake and listed in the records.

#### Demographic variables

Income was verified from official documents (e.g., pay stubs) during the intake visit at the WIC office to confirm eligibility criteria. Age was obtained using government-issued identification during the first WIC visit. Age was dichotomized into women less than 35 years old and women 35 years old and older. In this study maternal age was defined as the age at which the mother gave birth to the child. Maternal education was collected from medical records. Education was categorized into a three-level categorical variable (less than 12 years, 12 years, and more than 12 years). Nativity was obtained from vital records, which included exact information about the mother's country of birth.

#### Health variables

Health information was obtained from electronic medical records. We included health variables that have been shown to be independently associated with breastfeeding such as parity,^27^ body mass index (BMI),^28^ antenatal smoking,^29^ gestational age,^30^ and delivery method.^31^ Parity was dichotomized into first-time mothers (primiparas), and those who had had a previous live birth (multiparas). BMI was obtained from medical records and categorized into a three-level categorical variable for normal weight, overweight, and obese. Antenatal smoking was assessed through self-report during the first visit. Child birthweight was collected at the first postpartum visit using infant medical records. Gestational age was obtained through self-report during the first postpartum visit. Delivery method was self-reported during the first postpartum visit.

#### Antenatal depressive symptoms

Depressive symptoms were assessed through self-report using the EPDS. The EPDS is a standardized measurement of depression.^26^ For this study we used a cutoff of 10, which is what the local WIC office uses to detect all possible cases in highly diverse populations.^26,32^ Any report of elevated depressive symptoms during one of the trimesters was coded as elevated antenatal depressive symptomology.

### Data analysis

Chi-squared tests were used to detect any significant bivariate relationships between breastfeeding initiation and demographic, health-related factors, and antenatal depression. Only bivariate relationships of statistical significance (set at <0.05) were included in the logistic regression models. We conducted multivariable logistic regression to test the association between breastfeeding initiation and demographic and health-related variables. All the statistical analysis was conducted using STATA 14.2.^33^

## Results

The majority of participants were Black (40.10%), followed by White (38.51%). This study also included Latina (13.27%), Asian (5.54%), Multiracial (1.58%), and Other race (1.09%) women. The majority of the participants (91.68%) were under the age of 35. Thirty percent of women had less than 12 years of education, 37.82% had exactly 12 years, and 32.08% had more than 12 years. Sixty-six percent of study participants were born in the United States and 37.82% were primiparas. In terms of BMI, 40.79% of participants were in the healthy BMI range, while 24.55% of them were overweight and 31.98% were obese. The prevalence of antenatal depressive symptoms was 10.40% (Table 1).

**Table 1.** **Sample Characteristics of Participants by Breastfeeding Initiation**

Characteristic	Sample total *n*=1010 (%)	Initiated breastfeeding *n*=682 (%)	Did not initiate breastfeeding *n*=328 (%)	*p*
Race/ethnicity				
Black	405 (40.10)	221 (54.57)	184 (45.43)	<0.01
White	389 (38.51)	295 (75.84)	94 (24.16)	
Asian	56 (5.54)	52 (92.86)	4 (7.14)	
Latina	133 (13.17)	100 (75.19)	33 (24.81)	
Multiracial	16 (1.58)	6 (37.50)	10 (62.50)	
Other	11 (1.09)	8 (72.73)	3 (27.27)	
Education				
<12	301 (29.80)	173 (57.48)	128 (42.52)	<0.01
12	382 (37.82)	233 (60.99)	149 (39.01)	
>12	324 (32.08)	275 (84.88)	49 (15.12)	
Missing	3 (0.30)	1 (32.48)	2 (66.67)	
Age				
<35	926 (91.68)	614 (66.31)	312 (33.69)	<0.01
≥35	71 (7.03)	61 (85.92)	10 (14.08)	
Missing	13 (1.29)	7 (53.85)	6 (46.15)	
Median income				
<10,400	503 (49.80)	291 (57.85)	212 (42.15)	<0.01
≥10,400	507 (50.20)	391 (77.12)	116 (22.88)	
Nativity				
U.S. born	674 (66.73)	407 (60.39)	267 (39.61)	<0.01
Foreign-born	183 (18.12)	164 (89.62)	19 (10.38)	
Missing	153 (15.15)	111 (72.55)	42 (27.45)	
Parity				
Primiparous	382 (37.82)	283 (74.08)	99 (25.92)	<0.01
Multiparous	608 (60.20)	385 (63.32)	223 (36.68)	
Missing	20 (1.98)	14 (70.00)	6 (30.00)	
Method of delivery				
Vaginal	609 (60.30)	404 (66.34)	205 (33.66)	0.35
C-section	330 (32.67)	225 (68.18)	105 (31.82)	
Missing	71 (7.03)	53 (74.65)	18 (25.35)	
Body mass index				
Normal	412 (40.79)	294 (71.36)	118 (28.64)	<0.01
Overweight	248 (24.55)	176 (70.97)	72 (29.03)	
Obese	323 (31.98)	196 (60.68)	127 (39.32)	
Missing	27 (2.67)	16 (59.26)	11 (40.74)	
Antenatal smoking				
Yes	213 (21.09)	122 (57.28)	91 (42.72)	<0.01
No	794 (78.61)	558 (70.28)	236 (29.72)	
Missing	3 (0.30)	1 (33.33.0)	2 (66.67)	
Gestational age				
≥37 weeks	892 (88.32)	607 (68.05)	285 (31.95)	0.49
<37 weeks	107 (10.59)	67 (62.62)	40 (37.38)	
Missing	11 (1.09)	8 (72.73)	3 (27.27)	
Infant birth weight				
≥2500 g	909 (90.00)	617 (67.88)	292 (32.12)	0.70
<2500 g	97 (9.60)	62 (63.92)	35 (36.08)	
Missing	4 (0.40)	3 (75.00)	1 (25.00)	
Antenatal depressive symptoms				
<10	632 (62.57)	423 (66.93)	209 (33.07)	0.29
≥10	105 (10.40)	78 (74.28)	27 (25.71)	
Missing	273 (27.03)	181 (66.30)	92 (33.70)	

We also found a significant relationship between breastfeeding initiation and race/ethnicity (*p*<0.01), maternal age (*p*=0.002), education level (*p*<0.001), income level (*p*<0.001), nativity (*p*<0.001), parity (*p*<0.01), BMI (*p*<0.01), and smoking before pregnancy (*p*<0.01; Table 1). We did not find a significant relationship between breastfeeding initiation and gestational age, birthweight, delivery method, or antenatal depression. For parsimony, we included only relationships of statistical significance into the logistic regression models.

Table 2 shows the results of multiple logistic regression for predicting the variables of breastfeeding. In Model 1, only race/ethnicity was included. As shown in Table 2, Black (odds ratio [OR] 0.38; confidence interval [95% CI] 0.28–0.52) and multiracial (OR 0.19; 95% CI 0.07–0.54) women were less likely to initiate breastfeeding compared with White women. On the other hand, Asian women (OR 4.14; 95% CI 1.46–11.76) were significantly more likely to initiate breastfeeding compared to their White counterparts. There were no significant differences between Latina and White women. In Model 2, maternal age, education, income, and nativity were included. After controlling for these demographic variables, Black (OR 0.46; 95% CI 0.33–0.65), multiracial (OR 0.23; 95% CI 0.08–0.69), and Latina women (OR 0.49; 95% CI 0.27–0.87) were significantly less likely to initiate breastfeeding compared with White women. Moreover, Asian women were no longer significantly different from White women in terms of breastfeeding initiation. Also, it was found that women who were born in the United States (OR 0.19; 95% CI 0.10–0.36) were significantly less likely to initiate breastfeeding compared with those who were foreign-born. In terms of education, women who had more than 12 years education were three times more likely to breastfeed [95% CI 2.19–5.03], compared with those who had less than 12 years of education.

**Table 2.** **Summary of Logistic Regression Analysis Predicting Breastfeeding**

	Model 1		Model 2		Model 3	
Variable	OR	CI	OR	CI	OR	CI
Race						
Black	0.38^***^	0.28–0.52	0.46^***^	0.33–0.65	0.47^***^	0.34–0.66
White (reference)	1		1		1	
Asian	4.14^**^	1.46–11.76	0.76	0.24–2.47	0.64	0.19–2.10
Latina	0.96	0.61–1.52	0.49^*^	0.27–0.87	0.48^*^	0.26–0.86
Multiracial	0.19^**^	0.07–0.54	0.23^**^	0.08–0.69	0.21^**^	0.07–0.65
Other	0.85	0.22–3.27	0.62	0.14–2.77	0.69	0.15–3.10
Age						
<35 (reference)			1		1	
≥35			2.07	0.99–4.33	2.56^*^	1.21–5.44
Education (years)						
<12 (reference)			1		1	
=12			1.23	0.88–1.72	1.17	0.83–1.65
>12			3.32^***^	2.19–5.03	2.94^***^	1.92–4.50
Income						
<10,400 (reference)			1		1	
≥10,400			1.34	0.98–1.83	1.44^*^	1.05–1.98
Nativity						
Foreign-born (reference)			1		1	
U.S. born			0.19^***^	0.10–0.36	0.19^***^	0.10–0.37
Parity						
Primiparous (reference)					1	
Multiparous					0.60^**^	0.43–0.83
Body mass index						
Overweight					1.01	0.69–1.49
Obese					0.73	0.51–1.04
Smoking					0.83	0.59–1.17

^*^ *p*<0.05; ^**^p<0.01; ^***^p<0.001.

In the final model, parity, BMI, and antenatal smoking were included. The results from Model 3 showed that differences by race and ethnicity persisted. Black (OR 0.47; 95% CI 0.34–0.66), multiracial (OR 0.21; 95% CI 0.07–0.65), and Latina women (OR 0.48; 95% CI 0.26–0.86) were significantly less likely to initiate breastfeeding in comparison with White women. The effect of nativity remained; women born in the United States (OR 0.19; 95% CI 0.10–0.37) were less likely to initiate breastfeeding compared with their foreign-born counterparts. Moreover, it was found that women who were above the median income (OR 1.44; 95% CI 1.05–1.98) were more likely to initiate breastfeeding. Women with more than 12 years of education remained significantly more likely to initiate breastfeeding compared with those with less than 12 years. In addition, women who were 35 and older were 2.6 times more likely (95% CI 1.21–5.44) to initiate breastfeeding compared with participants who were under 35 years of age. A final important finding was that multiparous women were less likely to initiate breastfeeding (OR 0.60; 95% CI 0.43–0.83) compared with primiparous women.

## Discussion

This study investigated the differences in breastfeeding patterns among six different racial/ethnic categories to better understand the correlates and predictors among a sample of WIC mothers in a Midwestern county. Our analyses demonstrated that overall, 67.5% of WIC participants initiated breastfeeding, which does not come near the national average of 79%. Even more worrisome, this study found very different breastfeeding initiation rates by race/ethnicity; with only 54.6% of Black women and 37.5% of multiracial women initiating breastfeeding. After controlling for demographic and other characteristics, Black, multiracial, and Latina women were less likely to initiate breastfeeding compared with White women.

The results from this study are consistent with the literature that has shown breastfeeding initiation rates to be different by race/ethnicity. For instance, a study investigating racial/ethnic differences on breastfeeding behaviors (*n*=49,135) found that Latina women initiated breastfeeding at a higher rate (86%) than Black (64.2%) and White women (77.1%).^34^ Our rates for breastfeeding initiation among White participants in our study mirror those of Ahluwalia et al.,^34^ but they are lower for Black and Latina women. Another study comparing breastfeeding behaviors among White and Black WIC and non-WIC participants in Mississippi found that breastfeeding initiation rates were about 12 percentage points lower for Black women (38.4%) compared with White women (50.8%).^19^ Although the present study also found different initiation rates by race, the breastfeeding initiation gap was wider for Whites compared with Blacks and multiracial women than previously found. These considerable differences in breastfeeding initiation rates among WIC participants are alarming because they can influence the development of health issues among children and women in the future.^35^

Regarding Latino ethnicity, this study found discrepant findings among Latina women. In the unadjusted model, Latina women did not have significantly different breastfeeding initiation patterns compared with White women. However, after controlling for demographic factors, Latina women had significantly lower breastfeeding initiation rates compared with White women. A possible explanation for these findings is that nativity and the cultural norms that go with it play an important role when deciding whether to initiate breastfeeding. Previous research has demonstrated that foreign-born women are more likely to breastfeed compared to their native-born counterparts, irrespective of race/ethnicity.^36,37^ Other studies specifically conducted among Latina women have shown that lower levels of acculturation are associated with higher rates of breastfeeding initiation,^34,36,38,39^ suggesting that nativity and the cultural norms of the native country play a decisive role in the decision to initiate breastfeeding. These findings underscore the importance of continuing to investigate the role of nativity not only on breastfeeding initiation, but also on duration and exclusivity. Moreover, qualitative studies are necessary to identify the cultural components that facilitate or hinder breastfeeding initiation among native and foreign-born women.

Contrary to previous studies that have found antenatal depression to be associated with decreased likelihood of breastfeeding,^23^ this study did not find a relationship. It is possible that we did not find this relationship because the local WIC program offers depression management to women with elevated depressive symptoms. Another reason is that depressive symptoms were measured at only one time point during the antenatal period (i.e., we did not have depression measures for all three trimesters). As such, these symptoms could be a reaction to stressful life events (and resolve) rather than a depressive disorder. It can also be possible that the role of antenatal depression on breastfeeding does not have to do with initiation, but rather with duration, as some recent studies have shown.^40^ Nevertheless, it is necessary to continue investigating the role of antenatal depression on breastfeeding and provide women who desire to breastfeed with increased support when elevated depressive symptoms are present.

An important finding of this study was that primiparous women were more likely to initiate breastfeeding, compared with multiparous women. Previous research on the association between parity and breastfeeding is still unclear. One of the few studies that has investigated parity and breastfeeding found that multiparous women were less likely to breastfeed in the hospital (adjusted OR 0.67; *p*<0.05) compared with primiparous mothers.^41^ Our study mirrors Grummer-Strawn's previous findings by demonstrating that multiparous women were less likely to initiate breastfeeding compared with primiparous women.^41^ It is possible that women who have had previous experience with breastfeeding already know whether or not they want to breastfeed their next children and might be less inclined to attempt to breastfeed, compared with primiparous mothers, who are experiencing breastfeeding for the first time. In fact, a recent study demonstrated that multiparous women had very different breastfeeding experiences compared with primiparous women.^27^ Multiparous women reported fewer breastfeeding problems and had longer breastfeeding duration compared with primiparous women.^27^ These findings suggest that it is imperative to provide enough resources and support to women who decide to breastfeed for them to have a satisfying experience, which in turn could promote future breastfeeding.

A unique aspect of this study was the inclusion of multiracial women. Previous research investigating differences on breastfeeding by race/ethnicity has often overlooked health behavior patterns among multiracial women. Therefore, this study aimed to address this gap by providing initiation rates for this growing population. Including information on multiracial women is imperative given that previous studies have shown that multiracial people fare worse than their monoracial peers.^42,43^ In this study, we found that multiracial women have the lowest breastfeeding initiation rates, compared with women of any other racial/ethnic group. To address racial/ethnic disparities, it is necessary that studies conducted in the future include information on multiracial participants and compare their health behaviors and outcomes to other racial/ethnic groups. Moreover, it is necessary to develop breastfeeding interventions that focus on promoting breastfeeding and addressing the needs of Black, multiracial, and Latina women.

This study had numerous strengths that should be considered. First, this study included a large sample size and a diverse pool of participants, which allowed us to investigate breastfeeding initiation behaviors among racial groups that are usually not included in the analysis. Second, our study adjusted for a number of demographic and health characteristics that have been shown to be associated with breastfeeding behaviors.

This study also had some limitations. First, as mentioned before, it is possible that the support that is offered at the local WIC office masked the effect that antenatal depression can have on breastfeeding initiation. The support offered for breastfeeding and depression included close follow-ups, referrals to mental health services, support groups, and appointments with a group of diverse lactation consultants, among others. A second limitation was that initiation rates among Latina women were not separated by ethnicity. We understand the Latino population is heterogeneous and breastfeeding initiation rates have been found to be higher among Mexican and Mexican American women and lower among Puerto Rican women.^44^ Unfortunately, given the small sample size of Latina women in our study, we were unable to further reduce the group by ethnicity. Lastly, because the participants for this study were drawn from a depression registry, the findings regarding breastfeeding may not be generalizable to nondepressed populations.

## Conclusion

Breastfeeding initiation was found to be different among racial/ethnic groups. Black, multiracial, and Latina women were less likely to initiate breastfeeding compared with White women. Even after controlling for demographic and health characteristics, racial/ethnic differences on breastfeeding initiation remained significant. Antenatal depression was not found to be associated with breastfeeding initiation, although this could be the result of strong professional support for depressed mothers. Based on these results, breastfeeding promotion and support should remain at the forefront of maternal and child health promotion, with a particular focus on racial and ethnic minorities, to reach the *Healthy People 2020* breastfeeding goal.

## Abbreviations Used

BMI: body mass index
CI: confidence interval
EPDS: Edinburgh Postnatal Depression Scale
OR: odds ratio
SES: socioeconomic status
WIC: Women, Infant, and Children
